# Pulmonary Hypertension: Scientometric Analysis and Density-Equalizing Mapping

**DOI:** 10.1371/journal.pone.0169238

**Published:** 2017-01-04

**Authors:** Michael Götting, Mario Schwarzer, Alexander Gerber, Doris Klingelhöfer, David A. Groneberg

**Affiliations:** 1 Division of Health Economics and Metrics, Institute of Occupational Medicine, Charité-Universitätsmedizin Berlin, Free University Berlin and Humboldt-University Berlin, Berlin, Germany; 2 Institute of Occupational Medicine, Social Medicine and Environmental Medicine, Goethe Universität Frankfurt, Frankfurt, Germany; Kurume University School of Medicine, JAPAN

## Abstract

Pulmonary hypertension (PH) is characterized by the increase of the mean pulmonary arterial pressure in the lung circulation. Despite the large number of experimental and clinical studies conducted on pulmonary hypertension, there is no comprehensive work that analyzed the global research activity on PH so far. We retrieved the bibliometric data of the publications on pulmonary hypertension for two periods from the Web of science database. Here, we set the first investigation period from 1900 to 2007 (t1) due to the cited half life of articles and the relating difficulties to interpret the citation parameters. The second evaluation period (t2) covers the time interval from 2008 onwards including the year 2015. The data were analyzed and processed to density-equalizing maps using the NewQIS platform. A total number of 18,986 publications were identified in t1 that come from 85 countries. The US published the highest number of publications (n = 7,290), followed by the UK, Germany, Japan and France. In t2 19,676 items could be found worked out by 130 countries. The raking started just the same with the USA as most publishing nation with 7,127 publications on PH, followed by the UK and Germany. Japan fell back on 6^th^ place, whereas China came into view on the 5^th^ position. Analyzing the average citation rate as a parameter for research quality, Mexico reached the highest value in t1 and Ireland in t2. While, the country specific h-index underlined the leading position of the US research in both evaluation periods again. The average number of international collaboration items was expanding from none in 1978 to 530 items in 2015 with the USA as the country with the highest number of collaboration articles. The present study is the first large scale density-equalizing mapping and scientometric analysis of global PH research activity. Our data draw a sketch of the global research architecture in this field, indicating a need for specific research programs in countries with a lower human development index.

## Introduction

Pulmonary hypertension (PH) is characterized by the increase of the mean pulmonary arterial pressure (mPAP) in the lung-circulation. Due to the chronically overload of the right ventricle the heart is affected by a PH and is often developing a chronic cor pulmonale which can lead to a right heart failure [1]. PH was previously classified into 2 categories: 1) primary pulmonary hypertension; or 2) secondary pulmonary hypertension according to the presence of identified causes or risk factors [2]. Since the second World Symposium on pulmonary hypertension (WSPH) held in Evian, in 1998, a clinical classification was established in order to individualize different categories of PH sharing similar pathological findings, similar hemodynamic characteristics and, similar management. In the following symposia (Venice—2003, California—2008) several modifications of treatment strategies were introduced caused by the reflection of the scientific progress. During 5^th^ WSPH in Nice in 2013 substantial changes of the classification and the treatment of PH has been adopted and some child specific items has been added [3–12].

Concerning the epidemiology of PH, a number of registries provided important information about epidemiologic and phenotypic features of PH. In this respect, changes in the phenotype were observed in the past decades, including changes in survival, age, sex, and comorbidities [13–17]. Despite the relatively young mean age of patients with idiopathic PAH in the first published registry with 36 ± 15 years [18], PAH is currently more frequently found in elderly individuals. This leads to current mean ages between 45 ±17 and 65 ± 15 years at diagnosis [13–17]. Importantly, the age of diagnosis in PH is different according to the group of disorders that cause PH. For example, in the REHAP registry, the age at diagnosis of pulmonary arterial hypertension was 45 ±17 years, however, patients with chronic tromboembolic pulmonary hypertension were diagnosed at age 61± 15 years.

Next to this change in epidemiologic features, also the pathophysiological concept of PH has changed from a solely vasoconstrictive to a vasoproliferative genesis recent years [19–22]. Thereby new pharmacological targets have been identified that have changed the therapeutic strategy [20, 23–27]. The current scheme of therapy is elegantly described in several recent reviews and consists of drug classes including prostaglandins, phosphodiesterase-5-inhibitors, endothelial-receptor blockers and guanylatcyclase-stimulators [28–35]. After failure of pharmacological treatments lung transplantation remains as final and difficult therapeutic option [36–38].

During the last years enormous research efforts have been undertaken to improve our knowledge of the pathophysiology of the disease in order to provide new options for treatment and diagnostics. However, the enormous amount of data has not been analyzed in a profound manner concerning global research activities as in other fields [39]. Therefore, PH was included to the international study project “New quality and quantity indices in science” (NewQIS) [40, 41]. It was the aim of the present study to combine advanced density-equalizing algorithms and scientometric tools to draw a sketch of the global PH research architecture in the defined period between 1900 and 2007.

## Methods

### Data source

Data was retrieved from the Web of Science (WoS) database that is provided by the Thomson Reuters Institute for Scientific Information (ISI). The original data is publicly accessible via the link: http://www.med.uni-frankfurt.de/institut/arbeitsmedizin/Public-Repository/PulHypertension/index.html*

### Search strategies

In order to approximate the overall number of publications on pulmonary hypertension the following phrase was entered in the search field: „pulmonary hypertens*”OR „pulmonary arterial hypertens*“. The Boolean operator ‘OR’ joined the terms together, the asterisk (*) was used to cover all possible word endings such as hypertension, hypertensive or others. Due to the cited half life of articles that set the maximum of biomedical citations approximately at five to eight years after their publication, the analyzed first time span (t1) included the period from 1900 to 2007. The following period covered the time between 2008 and 2015 (t2) to assess the more current developments of the publication outcome.

### Citation quantities

The findings from the search process were analyzed with the ‘citation report’ function to determine the citations per year as well as the modified h-index calculated in a PH-specific manner and adjusted also to the publishing countries. Additionally, the citation rate which is the sum of the total times cited divided by the number of items in the set, has been calculated and analyzed.

### Data categorization

The bibliometric data from the search process results were downloaded in units of 500 publications by using the ‘output records‘ and ‘save as plain text’ functions from WoS. The digital information was transformed into an Access-Database format and the items were software-controlled analyzed for the origin of the publications by using the author address as the criterion for the publishing country. Furthermore, the data files were examined concerning a variety of different parameters e.g. the publication date, the authors and the publishing journals.

### Density-equalizing mapping

Density equalizing map projections (DEMP) were calculated and generated for this study [42]. In brief, to visualize the distribution of the overall number of published items and the average citation rate in a country specific manner, density-equalizing mapping procedures were calculated using the algorithm developed by Gastner and Newman [43]. In these calculations, the area of each country was scaled in proportion to a particular variable, i.e. the number of published items. This resizing procedure of the countries leads to a modified and distorted global map presenting the geographic allocation of a specific parameter.

### Quality criteria

To evaluate both, quantity and quality of a publishing institution, a modified version of h-index was established. In brief, the h-index of *h* can be calculated, if *h* of all publications received at least *h* citations each [44]. The index, normally used for the author’s overall scientific output, refers in this study only to the PH-specific publications and has been employed on the assessment of the publishing countries.

The Impact-Factor is defined as the average number of citations per year given to those publications from an institution, that were published during the two preceding years [45]. The Impact-Factor was used to compare the most productive journals among each other.

### Analysis of cooperation

To evaluate the international cooperation in publications, the data from the created Access-Database were software controlled analyzed to differentiate between cooperation-publications and single-country articles. The isolated publications from international cooperation were analyzed regarding the quantity of the cooperation between every single publishing country and another.

## Results

### Analysis of origin

**Fig 1:**
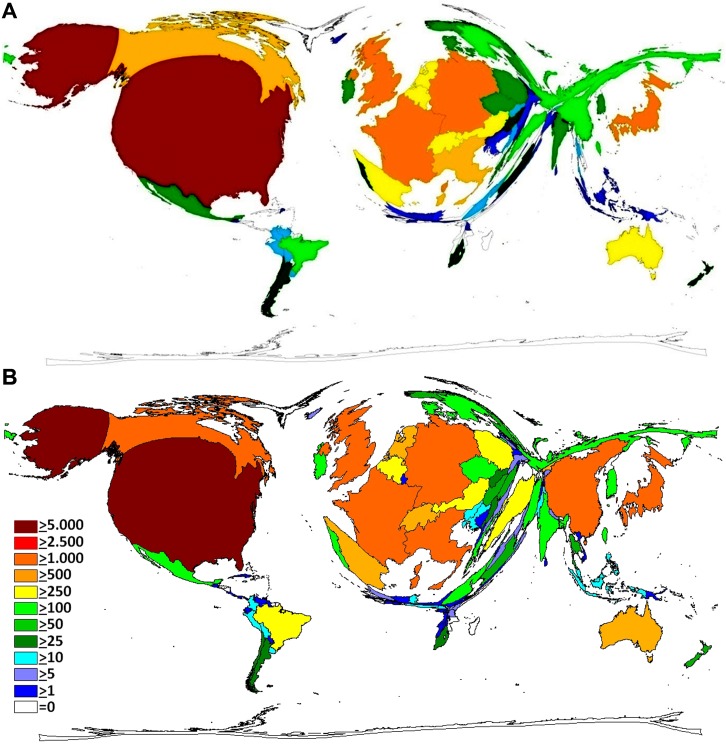
Density Equalizing Map Projection, Publication output. A) 1900–2007 B) 2008–2015, Legend = number of publications.

During the first evaluation period from 1900 to 2007 (t1), 18,986 published items (n) were worked out and included in the Web of Science database. They originated from 85 countries with the USA being the most prolific one having contributed in 40,7% (n = 7,290) of the total amount. The UK, Germany, Japan and France had more than 1,000 publications. The cumulated publications of the top five countries encompassed 67,8% of all indexed articles, taking into account that 1,396 publications were international collaborations and 2,907 items could not be assigned to a certain country (15.3%). The DEMP of this set of data visualizes the USA being responsible for the majority of the research efforts by far (Fig 1A).

From 2008 to 2015 (t2) a similar picture can be shown (Fig 1B). The USA published also in this time interval the most items with a contribution in 36,2% (n = 7,127) of the 19,676 items. The top five countries achieved even 72.9% of the overall publications. The international collaborations resulted in 3,442 items. Only 3.4% could not be assigned to a specific country in t2.

**Fig 2:**
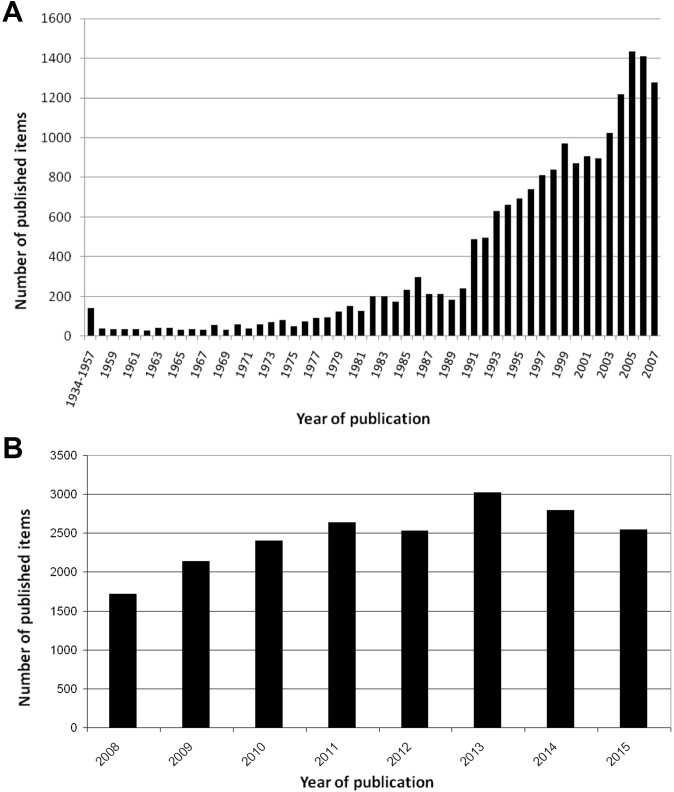
No. of PH-specific publications over the time. A) 1900–2007 B) 2008–2015

The first Article was published in the year 1934 but in the following years the output was inconstant, only 143 items were published until the year 1958. From that time on the numbers of items steadily increased until a peak was reached with 1,432 publications in the year 2005. The annual increase was interrupted by intermittent drops of the number of published items in 1987–1990 and 2000–2002 (Fig 2A). During the period from 2008–2015 the figures raised more from 1,702 to 3,011 articles in 2013. In the last two years of the evaluation period a small decrease to 2,533 items could be stated (Fig 2B).

### Citation parameters

**Fig 3:**
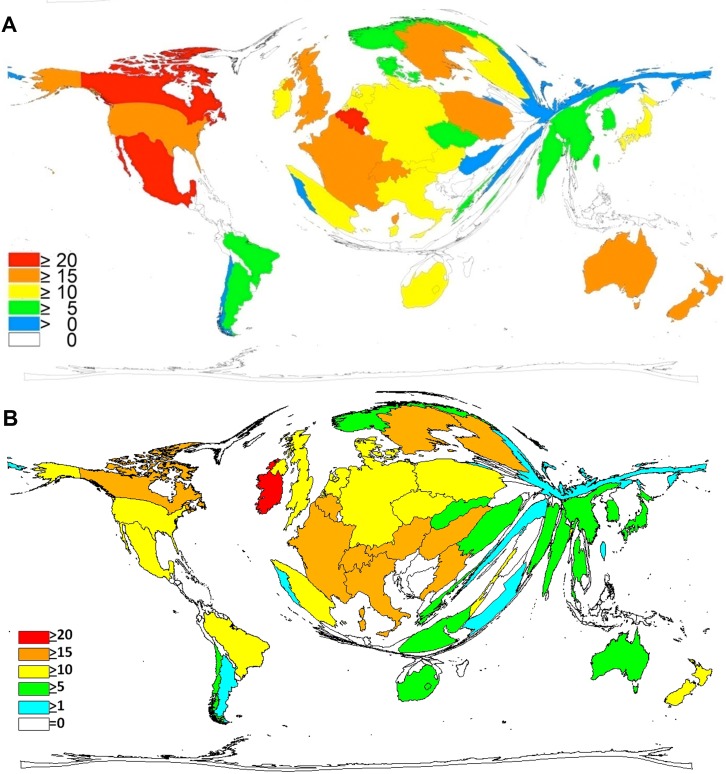
Density Equalizing Map Projection, citation analysis. A) 1900–2007 B) 2008–2015, Legend = citation rate.

To determine the research quality of the countries the average number of citations per item was used as criteria for quality. For visualization, the DEMP technique was used to display citation rate (cR), with a threshold of at least thirty publications per country. When analyzing all country-specific articles in t1, Mexico had the highest citation rate (cR = 23.29), Canada ranked second (cR = 20.69) and Belgium third (cR = 20.41). The most productive country, the USA achieved only a value of 18.12 and assumed so only position 23. Europe’s leading countries, the UK (cR = 18.71), Germany (cR = 13.54) and France (cR = 18.35) occupied the same sequence as with the number of published items (Fig 3A).

In t2 (2008–2015) Ireland achieved the highest value with cR = 25.71. Second ranked Hungary (cR = 19.51) and the third place claimed Sweden (cR = 18.1). The European countries that reached high values apart from that are Switzerland (cR = 17.73), and Belgium (cR = 17.72). The USA–as in t1 –had not a leading position and attained only a cR of 11.56. Mexico dropped to an even lower position with a cR = 11,16 (Fig 3B).

In comparison to the research output quantity as showed in Fig 2, big differences in the apportionment of the surface area in proportion to the average citations per item of each country can be visualized (Fig 3).

**Fig 4:**
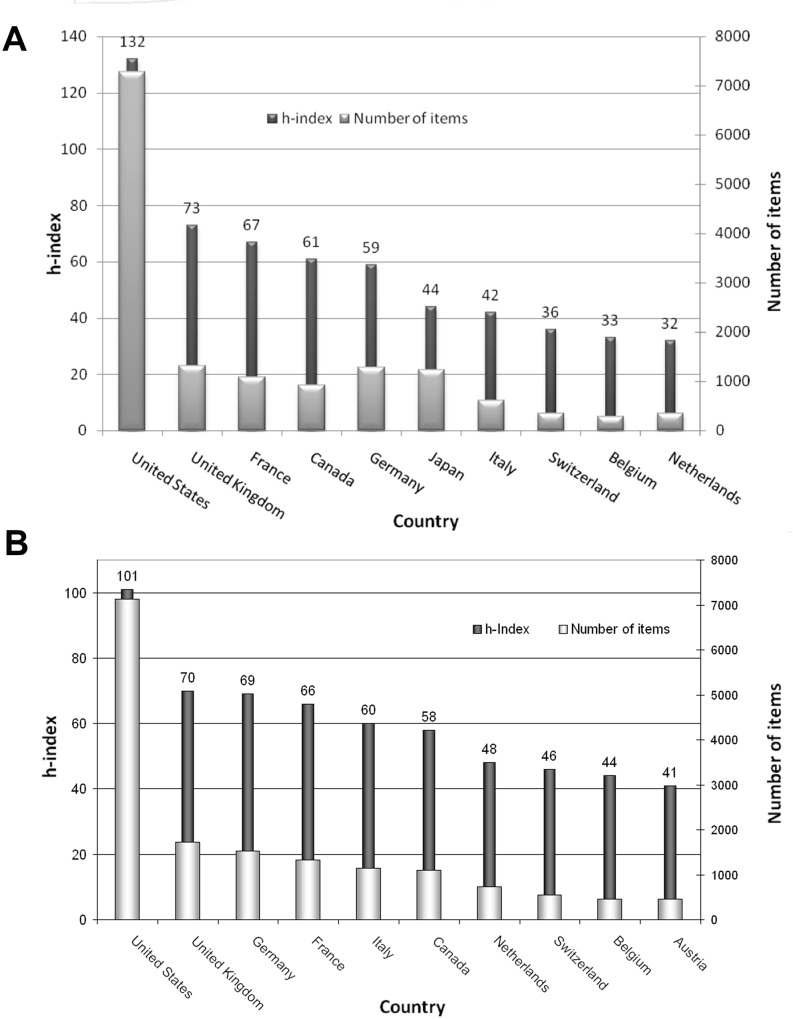
Modified h-Index of the most publishing countries. A) 1900–2007 B) 2008–2015

As a further parameter for the research quality of the publishing countries, modified the h-index (hI) was calculated for each country. With the highest modified index by far (hI = 132) in t1 (Fig 4A), the USA ranked first followed by the UK (hI = 73) and France (hI = 67). In t2 the two leading positions were also claimed by the USA (hI = 101) and the UK (hI = 70). Germany followed on the third position (hI = 69), whereas in t1 it reached only the 5^th^ position (hI = 59). France could be placed 4^th^ (hI = 66) in t2 (Fig 4B).

### Cooperation analysis

**Fig 5:**
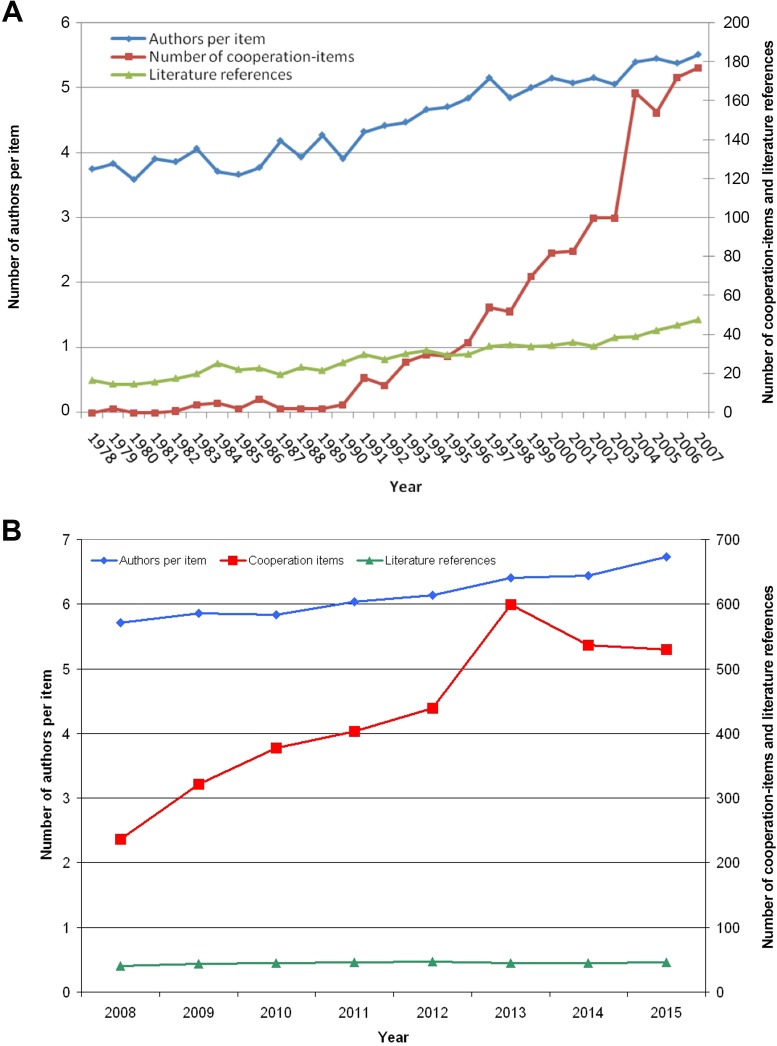
No. of authors per publication, no. of cooperation articles and no. of references over the time. A) 1900–2007 B) 2008–2015

The analysis of average number of international collaborations show that they grew from none in the year 1978 to 177 items in the year 2007 (t1) and furthermore, from 236 collaborations in 2008 to 530 items in 2015 (t2). In particular, the abrupt rise of collaborations between the years 2003 and 2004 from 100 to 164 and the high number in 2013 are remarkable (Fig 5A and 5B).

Additionally, the international collaboration network was reviewed and the number of collaborations for each country was calculated. In both evaluation periods, the USA was the country with the most research partnerships and the largest number of collaboration articles (cA). In t1 the strongest partner of the USA was Canada with 164 USA/Canadian collaborations, followed by the USA/UK (cA = 140), USA/France (cA = 110) and USA/Germany (cA = 109). In total, the USA collaborates with 54 different countries in an amount of 1,139 collaboration articles.

In t2 the strongest partner of the USA was the UK with an amount of 372 collaboration articles, followed by USA/Germany (cA = 321), USA/Canada (cA = 313) and USA/Italy (cA = 262). The USA worked out a total of 3,262 collaboration articles with 93 different countries in t2.

**Fig 6:**
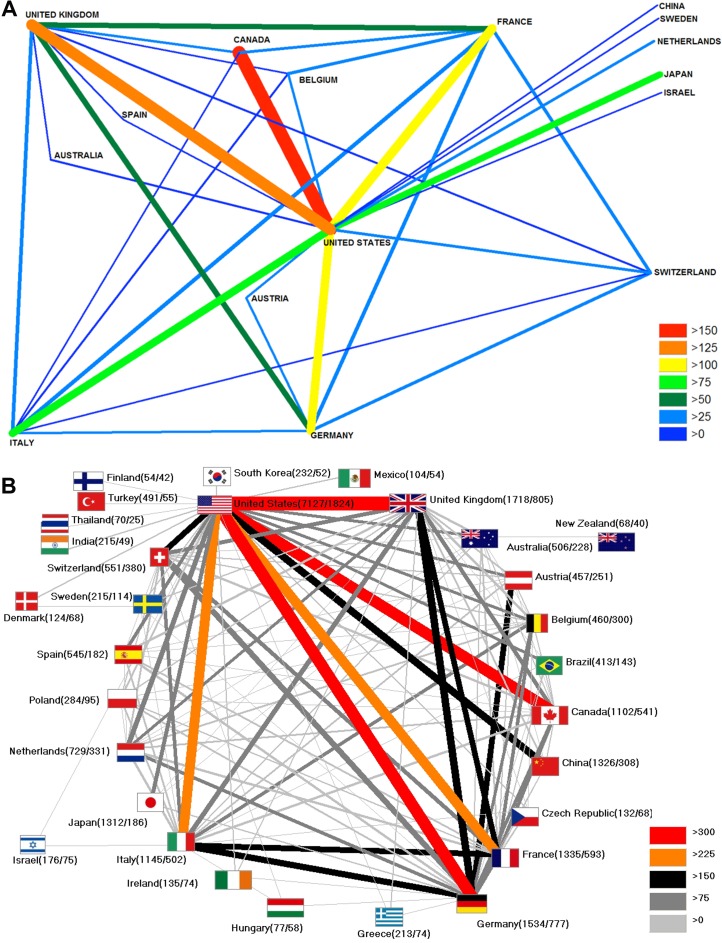
International collaborations. A) 1900–2007 B) 2008–2015, Legend = No. of collaboration articles, threshold: 20 items per country, colors and bar thickness encode amount of cooperation, numbers in brackets (publications / collaboration articles).

The spider charts in Fig 6 illustrate the international networks of both investigation periods from a threshold of 20 collaboration articles at least. Even at first glance, it can be recognized that the total amount of collaboration with more than 20 joint works has increased substantially in t2 (Fig 6B).

**Fig 7:**
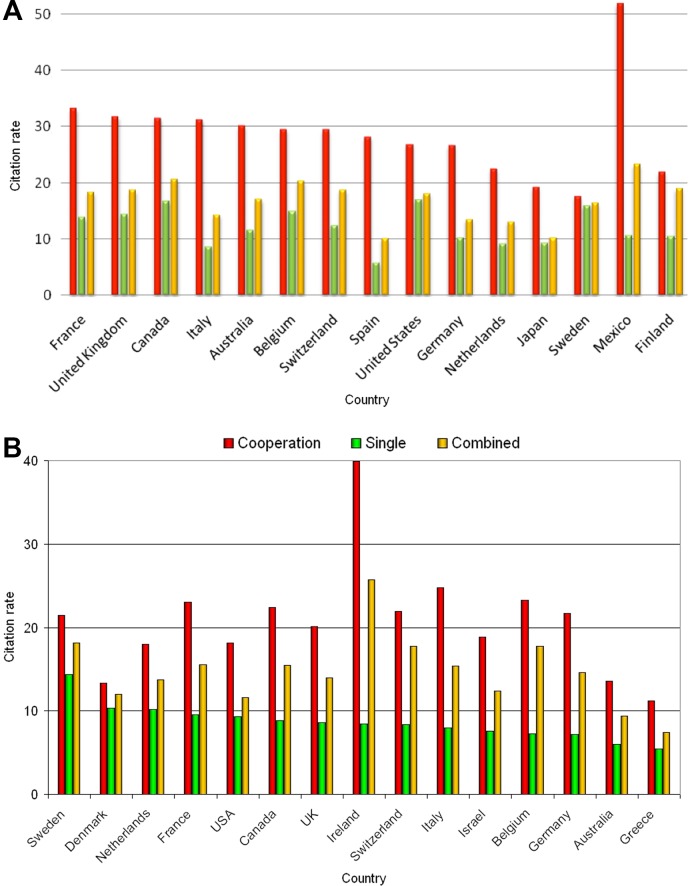
Country specific citation rate for single country articles (single), collaborations (cooperation) and all published items (combined). A) 1900–2007 B) 2008–2015

To differentiate between the publication output of the countries worked out together with other countries and the individual achievement of each country, the citation rate (cR) for the cooperation and single-country items as well as the combined accomplishment has been determined (Fig 7). The analysis of the fraction of the top countries regarding single-country items, cooperation-items and the combined value of the average citations of the different forms of publications show, that the well cited cooperation-items of Mexico in t1 (cR = 51.94) were responsible for the high value in combined-items (cR = 23.29). Concerning the citation rate for the single-country publications, the USA showed the highest rate (cR = 17.03) followed by Canada (cR = 16.74) and Sweden (cR = 15.96) in t1 (Fig 7A).

In t2 Ireland reached the highest citation rate for the cooperation items that are–similar to Mexico in t1 –responsible for the high overall citation rate of the combined values. Sweden received the highest rate for the individual country output (cR = 14.33), followed by Denmark (cR = 10.30) and the Netherlands with cR = 10.18 (Fig 7B).

### Analysis of the number of references and participating authors

Furthermore, the number of references and the number of the participating authors per published item were determined over the time. The average number of authors per item rose in the first period t1 from initially 3.75 in the year 1978 to 5.52 in 2007 (Fig 5A) and in t2 from 5.72 to 6.73 (Fig 5B).

In the same mode, the average number of quoted references per published item increased. Starting in 1978 with 16.46 references, the number duplicated to more than 47.76 in 2007. This level had not been reached in t2 with a slight increase from 40.7 in 2008 to 45.54 in 2015.

## Discussion

During the past decades, medical investigation has been spurred by a number of revolutionary new insights into pathogenetic and pathophysiologic mechanisms of pulmonary hypertension that led to significant changes in the management of the disease. However, despite these advantages in science and clinics a precise scientometric analysis of research activity has not been performed so far. Therefore, the NewQIS project elected PH as a research focus and performed a combined density-equalizing and scientometric study.

By the selection of WoS as data source a methodical bias cannot be excluded. Not all journals publishing on PH are listed. Particularly publications of national associations or recently founded journals have not found entry into WoS. Additionally, a majority of English-publishing journals can be found, so that a preference for these journals can be stated. Therefore, English-speaking countries should have an advantage.

A total of 38.662 publications related to PH were identified between 1900 and 2015 with a progressive increase since the beginning of the 1980’s and an abrupt rise from the year 1992 on. The maximum was reached in 2013 with 3011 publications. In principle, this picture reflects the usual tendency regarding the development of publication output in the biomedical sciences as shown in previous studies [46–48]. Additionally, this development might be used as indicator for an augmented interest in PH. On the one hand, great scientific advances such as the identification of nitric oxide as a vasodilative agent, the direct inhalation of nitric oxide (NO) for selective pulmonary vasodilation and the first studies on endothelin-1 antagonists should be mentioned in this respect [49, 50]. Furthermore, it can be assumed that major political health events like the 3rd WSPH in 2003 had an impact on the PH-research output [11, 51]. The high values in 2013 derive certainly from the importance of the 5^th^ WSPH held in Nice that delivered new aspects on PH leading to important changes regarding its treatment and classification [12].On the other hand, the steep increase in 1991 can be technically explained by the implementation of abstracts and keywords in WoS [52].

85 countries had been publishing on PH until 2007 and 130 from 208 onwards. The USA as leading country published the most articles on PH in both evaluation intervals (t1: n = 7,290; t2: 2,127). Only the combined publication output of Europe countries achieved a comparable amount. A relatively small number of countries was responsible for the majority of research on PH. The top three ranking of US, UK, Germany in t1 is not a unique pattern of PH research [53, 54]. It is also visible in numerous other respiratory fields [47] as well as in cardiologic fields [55, 56], in infectious diseases [46, 57], or public health issues [58, 59]. Until 2007 (t1) Japan and France followed the first three countries, whereas in t2 France and China occupied the next places, that means that China replaced Japan in ranking of the top five. This is certainly caused by the development of the Chinese economy in the last three decades, during which China has steadily increased their expenditures for research and development (R&D) and funded many scientific programmes that had also an enormous impact on the overall research output. Even in 2012 China invested nearly as much in R&D as Europe. The OECD estimated that China will be the country with the highest R&D expenditures in 2019 worldwide [60].

The leading position of the USA is underlined by the analysis of the international networks. The USA is involved in the majority of collaborations and at the same time the closest partner for many countries. By contrast, regarding the country’s citation rates, Mexico showed the highest value due to few high cited cooperation articles in t1. Ireland achieved the highest rate in t2. Here, undoubtedly their high cited collaboration work in relation to their overall publication output also accounts for. The University College of Dublin took part in the second most cited article on the alterations of the clinical classification in the aftermath of the 4^th^ WSPH in 2009 among nine other countries. This article titled “Updated Clinical Classification of Pulmonary Hypertension” by Simonneau et al. [61] has been cited 969 times alone at the time of retrieving the articles. The most cited article (1,337 citations) has been published by Galie et al. from the University of Bologna (Italy) also in 2009 “Guidelines for the diagnosis and treatment of pulmonary hypertension” [62]. This contributes definitely to the Italian’s placement among the best ten countries regarding their citation rate in t2.

The application of the PH-specific h-index, unlike the citation rates, diminish the overvaluation of few or single outstanding articles. This was confirmed by the findings that dies not put Mexico, respectively Ireland, among the highest ranked countries in this respect. Here, the USA again achieved the highest value in both evaluation periods.

While comparing the two evaluation periods, a trend towards a higher number of collaborations and the involvement of more nations worldwide can be stated. This underlines the importance of the global networking and its positive consequences [63].

In summary, the present study provides an in-depth scientometric analysis of the publications on PH since 1900 till 2015 in two separate time intervals, so that we can determine, examine and discuss changes in the publication activity. Regarding the research output, since 1934 a progressive increase could be shown that lead to an equally increase of citations. Using DEMP algorithms, a picture of the global PH research architecture was generated. The data indicates a need for specific research programs in countries with a lower human development index.
